# Availability of support systems for youth who left child and youth care centres during COVID-19

**DOI:** 10.4102/hsag.v30i0.2745

**Published:** 2025-01-25

**Authors:** Fadzaishe B. Zingwe, Maditobane R. Lekganyane

**Affiliations:** 1 Department of Social work, College of Human Sciences, University of South Africa, Pretoria, South Africa

**Keywords:** child and youth care centres, COVID-19, social work, support systems, youth

## Abstract

**Background:**

Youth who left child and youth care centres (CYCCs) during the coronavirus disease 2019 (COVID-19) were exposed to unique scale of challenges compared to their peers. Their transition period required them to have solid plans for their uncertain future towards independence. In further compounding their challenges was their being indeterminate given the uncertainties associated with COVID-19. It was therefore, essential for them to have the necessary support systems to aid them both during their period in care as well as when they left these centres.

**Aim:**

This study sought to explore available support systems for youth who left care during the COVID-19 pandemic.

**Setting:**

The setting of this study was Ekurhuleni metro municipality in Gauteng province of South Africa.

**Methods:**

This was a qualitative study. A total of 12 youth who left care centres during the COVID-19 were recruited through purposive sampling to participate in semi-structured interviews. Data analysis followed Braun and Clarke’s thematic analysis strategy, and trustworthiness was guided by credibility, dependability, transferability and confirmability.

**Results:**

The findings revealed the existence of support from various systems including the CYCCs themselves, social workers from both the CYCCs and the communities, sponsors, primary caregivers as well as friends.

**Conclusion:**

Youth who leave CYCC develop relationships from the care centres. These relationships form the basis of their support, upon which they rely even after their departure from these centres.

**Contributions:**

The study highlighted the importance of considering the impact of COVID-19 on the CYCCs and significance of supporting youth leaving them during pandemics such as COVID-19.

## Introduction and background

Traditionally, relatives and communities took on the role of caring for children, but these systems have come under tremendous pressure, leading to the placement of children and youth at the child and youth care centres (CYCCs) (Van Breda & Frimpong-Manso 2020). These CYCCs, which serve as a home to these children and youth, provide them with an opportunity to grow up in a supportive, home-like environment (Molano, Espinosa & Leon 2024). They play a crucial role in protecting children and youth who experience various challenges by setting a foundation for children to realise their potentials later in their lives (Cappa et al. 2021). Some of the reasons for children to be placed in these centres include neglect, abuse or abandonment by their own primary caregivers (Malatji & Dube 2017). Globally, around 2.7 million youth and children, were placed in CYCCs during 2017 (Cappa et al. 2021).

Within the South African context, a CYCC is a facility providing the court ordered residential care to more than six children outside their family environments (South African Government 2019). With the social challenges affecting most of the country’s families, the CYCCs became the most common alternative to issues affecting children and youth. Despite great initiatives such as CYCCs, child welfare practitioners and policymakers experience challenges when responding to complex and diverse issues faced by vulnerable children and youth. Although some studies have been conducted around the subject of CYCCs as well as the challenges faced by youth when leaving these centres, very few investigated availability of support systems for those who left these CYCCs during the coronavirus disease 2019 (COVID-19) pandemic. It was this research deficit that prompted the researchers to explore the availability of support systems for these youngsters when they leave care.

### Background on the coronavirus disease 2019 pandemic

Coronavirus disease 2019 is an infectious disease caused by the coronavirus that was discovered in Wuhan, China, in December 2019 (World Health Organization [WHO] 2020; Zhu, Wei & Niu 2020). The virus is spread primarily from person to person through small droplets from the nose or mouth expelled during coughing or sneezing. A person risks catching the virus if they inhale these droplets or touch surfaces where the droplets have landed (WHO 2020; Zhu et al., 2020). Situations that aggravate the spread of COVID-19 include mass gatherings, travelling and living in population dense areas.

The economy also suffered the impact of COVID-19 because key economic areas such as the aviation and tourism became suspended while some companies closed up due to unaffordable labour costs. Between 71 and 100 million people were estimated to be in extreme poverty because of COVID-19 (The United Nations Committee for Coordination of Statistical Activities 2020), and therefore exposed youth who became discharged during this period to poverty-stricken families. Socially, the pandemic affected the already marginalised and vulnerable people such as those with underlying conditions who were deemed to be susceptible to infection (Liu, Dean & Elder 2023; WHO 2020). Although all children were affected by the closure of schools, those who had no Internet access were at a greater disadvantage because educational institutions resorted to delivering lessons online (United Nations Committee for Coordination of Statistical Activities 2020).

#### Placement procedure for children and youth in South African child and youth care centres

Youth who find themselves placed in the CYCCs are mostly admitted as children and grow up in these centres. In terms of the South African practice, placing a child in CYCCs should be in the best interest of such a child and a last resort after considering various alternative placements such as temporary safe care and foster care (South African Government 2019). Section 150 (2) of the *South African Children’s Act* (hereinafter, the Act) provides that if a child is found to be in need of care and protection, a designated social worker must investigate their circumstances, specifically to determine the exact situation in which such a child finds himself or herself and to establish whether there is someone else close to the child who is willing to assume responsibility for them (South African Government 2006). The social worker must then proceed to present the findings of his or her investigation to a children’s court, which will then determine whether the child should be placed in a CYCC as prescribed by Section 158 (1) and (2) of the *Act* (South African Government 2006). The overall purpose of this process is to secure stability in the child’s life.

According to Section 156 (1) (e) (v) of the *Act,* if a child has no parent or caregiver or the former is there but is deemed unsuitable to take care of the latter, the court may issue an order placing the latter in a CYCC with residential care programme suitable to him/her (South African Government 2006). In order for a CYCC to admit a child into its facilities, the required documents include a valid court order issued by the children’s court, a medical examination report especially for those admitted as newborn babies, a clinic birth card, a social worker’s background report detailing the general socio-economic circumstances of the child, psychological assessment report issued by a psychologist and school report for those who are placed in care centres when they are already of school going age.

During 2014, a total of 11 105 children were placed in CYCCs in South Africa (South African Government 2019). These children are not placed permanently in these centres. They have to leave at a certain point of their lives and mostly do so as youth. By its international definition, youth is a period of transition from childhood to adulthood, which is characterised by independence (United Nations Youth, 2013). In South Africa, most of the youth remain in care until the age of 18, which is deemed to be the beginning of independence and transition into adulthood (Glynn 2021). However, in section 176(2) of the *Act* provides, for these young adults to remain in these centres even beyond the age of 18, on the condition that there is a caregiver who is still willing to care for them and that remaining in such a centre is crucial for the purpose of completing their education or training (South African Government 2006). The prolonged period that children spend in care often results in the establishment of relationships with the other children, members of staff and other people whom they meet during their period in these centres. These relationships are deemed essential in providing support both during the time the youth spend in these centres and when they have to leave (University of Kent 2023). Despite these relationships and availability of support systems from these centres, research evidence points to numerous challenges faced by youth when they leave these care centres.

#### The challenges encountered by youth in the child and youth care centres

When the time comes for youth to leave the care centres, they are presented with various challenges. For those who left these centres during the COVID-19 pandemic, the situation was even worse. They left at a time when there were unpleasant changes in society, which led to stress that increased their vulnerability, making it crucial for them to have the necessary support in order to become accustomed to life after care (Amissah 2021). Youth who leave care depend on networks such as parents, friends and other older adults within the community for advice, comfort and support throughout their adulthood (Doucet 2018). Some of these challenges are tied to their historical experiences, including their reasons to find themselves in these centres after all. Some children were removed from their biological families and placed in care when they were already adolescents, and they ended up struggling to adjust to life transitions in these centres. In such instances, they often exhibit signs of anger, frustration and behavioural issues because of being placed in these centres (Hoffnung-Assouline & Attar-Schwartz 2020). Another challenge faced by youth in care is the stigma associated with living in a care centre, mostly from their peers at school and fuelled by educators (Rogers 2017). A United Kingdom (UK) study by Rogers (2017) focussing on the experiences of youth in foster care for instance, revealed differential treatment by educators and other children when they found out that they were in foster care, with some peers and educators feeling sorry for them, which reinforced feelings of difference among youth. Some of the study participants reported being insulted by their peers for being in foster care, with insults often centred around a perceived rejection by their parents (Rogers 2017). When youth face challenges such as the above-stated, they require support to cope with such experiences.

#### Available systems to support youth in the child and youth care centres

The CYCCs take on the responsibility of supporting the youth to navigate through the challenges that they face. These centres strive to ensure that young people’s needs are catered for, including the physical, psychological, financial and emotional needs (Mamelani Projects 2015). Some youth find themselves in CYCCs as babies and the people at these centres become family to them, resulting in strong connections among them, which then makes the CYCCs to be their source of belonging. Belonging in this context refers to a positive feeling about one’s place within a supportive, beneficial system that extends beyond the individual’s self (The Children and Youth Planning Table [CUPT] 2021). This sense of belonging comes from the support that is provided through the care centres.

Among the professionals who play a central role in the CYCCs are social workers who render psychosocial support services by providing these youth with individual therapy and psychosocial group therapeutic intervention to enable them to better cope with issues that they face (Mamelani Projects 2015). Individual therapeutic interventions enable youth to express their deeper private issues more freely and in a private one-on-one interaction space with a trusted professional. Group therapeutic sessions present a platform for the youth to be supported and educated on the experiences that are common to them, such as the stigma that is faced by the youth at school. Another way through which CYCCs provide support is by arranging visits and telephone calls between the youth and their families so that they can maintain contact and work on issues that led to the youth’s placement in the centres (Harder et al. 2020).

It is important that CYCCs facilitate and encourage youth to develop close relationships with their families and other people so that they do not view the centre as their only source of support especially when they leave and to avoid waning ties with their families (Amissah 2021; Mamelani Projects 2015). In Andalusia, Spain, a programme called Collaborating Families was developed for the benefit of young people in care centres. The programme consisted of families who committed themselves to supporting children and youth by taking them on weekends and holidays to participate in alternative activities to those of the care centres (Molano et al. 2024). The Collaborating Families programme also encourages the children and youth to establish healthy bonds with the families and expand their support network in the process (Molano et al. 2024). In Denmark, a young person in care is allocated a ‘steady contact person’ whose role is to provide support and to determine whether he or she has a family or a wider network that can assist in providing stability even when such young person has left care (Boddy et al. 2019). All these measures are taken by care centres to support the children and youth.

#### Discharging youth from the child and youth care centres

In South Africa, youth are discharged from a CYCC following provisions of Sections 174 and 175 of the *Children’s Act No 38 of 2005*, when they reach the age of 18 and when they are no longer schooling or participating in any training programme (South African Government 2006). A discharge from a CYCC may also be because of reunification with the child’s immediate family. In some instances, they are discharged when the residential care programme at the CYCC is no longer benefitting them as per Section 172 (1) and (2) of *the Children’s Act No 38 of 2005* (South African Government 2006). A discharge because of a residential care programme not contributing to the development of a youth usually happens to those who are placed in care as babies, who then have to be discharged from one CYCC to the other because of the age group requirements of such a centre.

When youth leave care centres, they have to leave their old lives and start new ones, marking the beginning of the rest of their lives as adults. The prolonged period that they spend in care often results in the establishment of relationships with the other children, members of staff and other people whom they meet during their placement. These relationships often have an integral role of providing support both during their time in care and when they leave care (University of Kent 2023). Youth leaving care centres experience transition to adult life to be more complicated than those who are raised from their own family environments. Their challenges include dropping out of school, dealing with unemployment, experiencing financial insecurity, developing mental health issues, adapting to new environments outside the care centre and the challenges of reintegration into their families and communities (Haggman-Laitila, Salokekkila & Karki 2019). Although these challenges are generally common among youth during transition from care, they mostly became aggravated by the COVID-19 pandemic. For example, finding a job became more difficult during COVID-19 because some companies were retrenching their employees or shutting down because of the impact of COVID-19. Coronavirus disease 2019-related disruptions had the potential for lifelong implications on youth given their sensitive developmental phase (Gittings et al. 2021; United Nations’ Children’s Fund [UNICEF] 2020). Haggman-Laitila et al. (2019) point to youth transition as a multi-dimensional phase of personal development, which is shaped by their past experiences of vulnerability, the levels of support they have received and the challenges they face in becoming self-sufficient. There is a general consensus in the existing literature that COVID-19 set more hurdles for youth leaving CYCCs; hence, the need for support systems during this precarious period of their lives (Barford, Coutts & Sahai 2021; Modi & Kalra 2022; Munro et al. 2021).

Long (2022) focussing on SOS Children’s Villages reported that African countries such as Ghana, Zambia and Zimbabwe formed care leavers’ networks intended for youth to support one another and to ease the pressures that come with transition. These networks, which are led by youth who have exited care themselves, work by connecting with youth who have been in care centres on digital platforms such as WhatsApp, with the aim of addressing particular challenges faced by youth after leaving care, of which most were worsened by COVID-19 (Long 2022). Their main aim was to advocate to governments for change in polices and legislation pertaining to children in care centres and those who have exited these centres by accurately reflecting their needs (Long 2022). The formation of such networks is supported by UNICEF (2020) in their report, which suggested moderated WhatsApp groups for youth who have left care to establish buddy systems with their peers in order to check on each other’s well-being. An Irish study of peer relationships at school and in the children’s home by Emond (2014) also points to the shared experiences of having been in a care centre as a source of social support for the youth. This discussion highlights the necessity of support systems for youth when they exit care centres and when preparing those who are still in care for their exit.

### Theoretical framework of the study

**FIGURE 1:**
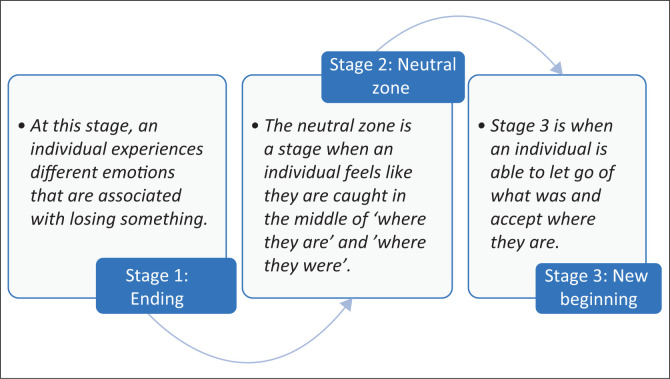
The transition model: William Bridges.

A theoretical framework is what informs and influences the researcher’s understanding of the social world (Bryman 2012). This study was informed by William Bridges’ Transition Model and the Systems Theory developed by Ludwig Von Bertalanffy. The model provides a framework for explaining different stages through which individuals go when they experience change or transition in their lives. According to Bridges’ Transition Model, transition is a process that evolves through three main stages: the Ending, the Neutral Zone, and the New Beginning (Leybourne 2016). The Transition Model helped to explain and enhance understanding of the different stages that the youth go through when leaving the care centres. Figure 1 demonstrates the process of transition from Bridges’ Transition Model.

Stage 1 of William Bridges’ Transition Model is the *Ending Stage,* and it is when an individual who is transitioning experiences losses as a result of letting go (Newbery 2019). The ending stage is actually the beginning of their transition, and it starts when people identify what they are losing and what they will keep (Newbery 2019). Youth who left the CYCCs had to leave behind the support that they used to enjoy in the form of accommodation, clothing and medical care. At this stage, they feel sad and afraid that they probably would not be able to manage life on their own without the support of CYCC workers, housemothers and friends (Leybourne 2016). During the ending stage, youth let go of the old identities, including that of being a youth in care who entirely depended on the care centre for support to an independent adult who is no longer entitled to support from the care centres (Miller 2017).

The *Neutral Zone* is stage two of the transition model, and it is the ‘in-between’ time, when the youth had left the centre, but their new experiences are not yet in full effect (Miller 2017). This is the stage when an individual will be trying to figure out where to go and how to move forward without the support that they were accustomed to having at the centre, often leading to low morale (Newbery 2019). Some of the participants of the study were still trying to figure out how they would move forward to pursue their education should sponsors withdraw financial support. Stage two is described as the ‘core’ of the transition process (Leybourne 2016). Confusion and distress are common feelings at this stage because individuals learn what their new roles will be and get acquainted to the support systems that are available for them.

Stage three of the model is the *New Beginning*, which involves new understandings, attitudes and values towards one’s situation. This is when one has a sense of direction, and their situation begins to make sense (Newbery 2019). The participants of the study made a new beginning at this stage and discovered a new sense of purpose, and some decided to go back to school after sponsors’ confirmations to continue supporting them by paying their fees and offering them accommodation (Miller 2017). Some got employment opportunities and they began to work. There were feelings of reorientation and renewal at this stage as participants realised that they still had support from people with whom they had built relationships when they were at the care centres.

Systems Theory on the other hand is an approach that seeks to understand human beings and their behaviour not in isolation, but in the context of societal factors and systems (Schirmer & Michailakis 2019). From the Systems theoretical perspective, the social environment and all of its influences enhance the understanding of the dysfunctionality or well-being of an individual (Davies 2022). As demonstrated further in this article, participants of this study were anxious about leaving care centres, where their needs were properly met. The relationships that they had with other youth and personnel in these centres was also a factor attracting their reluctance to leave. Davies (2022) contends that Systems Theory considers the effects of societal values on an individual, which could explain why the participants valued the relationships that they built during their time in care because they provided a sense of belonging. Systems Theory also brought enlightenment to the potential challenges that participants were to face in an unsupportive environment after leaving the care centres. Being in an unsupportive environment after leaving the centres would not have positive bearing on the youth, which is why care centres facilitated contact between youth and their families and resolved any pending issues prior to their discharge (Davies 2022). Each support system from friends to social workers, had an effect on how these youth managed transition.

While Bridges’ Transition Model contends that any individual going through a transition is likely to experience each stage of the process, the Systems Theory assesses how the connections that an individual makes during these stages of transition impact on support. Children and youth are vulnerable and they need support systems at each stage to develop better ways of leading positive lives.

## Research methods and design

The overall aim of this study was to explore the availability of support systems for youth who left CYCCs during the period of COVID-19. The main question that the study sought to answer was what kind of support systems are available for youth who left the CYCCs during the COVID-19 pandemic?

### The research approach and design

To answer this question, the study followed exploratory and descriptive qualitative research approach. Qualitative researchers assumed a perspective that suggests that truth is not absolute but rather contextual and multiple, valuing and acknowledging subjectivity. The interpretive, holistic and descriptive nature of qualitative research was particularly suitable to enable the researchers to comprehensively understand availability of support systems for youth from the subjective perspectives of the participants themselves (Creswell 2014; Leavy 2014). An exploratory research design involves the open-mindedness of the researchers in order to learn from the participants (Creswell 2014). Through the exploratory research design, researchers were in an opportune position to learn from the participants and explore their views. Akhtar (2016) considers the aim of descriptive research as to describe phenomena as they exist. It enables the researchers to portray the support systems that were available as described by the participants themselves.

### Research context

This study was conducted in South Africa’s city of Ekurhuleni, which is a Xitsonga name for ‘Place of Peace’. The place was named after its politically violent transitional period from apartheid to democracy, and it symbolises diversity (South African Government 2020). One of the reasons for considering Ekurhuleni for this study was the levels of poverty in the area, which was over 34.0%, unemployment at around 66.0% at the time of this study, with youth unemployment being at 36.9%, implying a strong dependence on state social assistance (i.e placement of youth and children in care centres) (South African Government 2020).

### Sampling, population and data collection

For participants to take part in this study, purposive sampling supported by the predetermined inclusion criteria were used. Eligible participants had to be: (1) youth who left care centres from the City of Ekurhuleni Metro Municipality of South Africa, during the COVID-19 pandemic, (2) between the ages of 18 and 23 years, and (3) be placed at a care centre for at least 2 years before being discharged. This selection process yielded a total of 12 participants.

For data collection, participants took part in semi-structured individual face-to-face interviews that lasted for approximately 60 minutes. As preferred by the participants, the interviews were conducted in English. Whereas the collected data were analysed following Braun and Clarke’s (2006) thematic analysis strategy, its trustworthiness was upheld through Guba and Lincoln’s classical principles of credibility, transferability, confirmability and dependability.

### Study rigour and trustworthiness

To achieve trustworthiness, the researcher used credibility, transferability, dependability and confirmability (Ahmed 2024; Stahl & King 2020). *Credibility* was upheld through prolonged engagement, triangulation and reflexivity, which involved spending time understanding the dynamics of the study site, using diverse literature and data sources (i.e. literature from different disciplines and interviewing participants from different centres). In achieving *confirmability*, researchers adopted peer debriefing, which involved seeking review from colleagues and member checking through which the findings were taken back to participants for confirmation of accuracy and correctness (Ahmed 2024; Stahl & King 2020). *Transferability* was realised through thick description of the methods, the process and the findings. For *dependability*, an audit trail accounting for decisions and procedures followed was adopted (Ahmed 2024; Stahl & King 2020).

### Ethical considerations

This study was guided by research ethical principles required for studies involving human participants. An application for full ethical approval was made to the University of South Africa’s College of Human Sciences Research Ethics Committee and ethics consent was received on 29 November 2021. The ethics approval number is 48087688_CREC_CHS_2021.

It was only after the ethical clearance was issued that the gatekeepers from CYCCs of the city of Ekurhuleni were contacted to approve access to study sites and the participants. As required by informed consent, participants were informed of the nature of the study, the researcher’s expectations as well as their right to withdraw from the study. Confidentiality was ensured by conducting interviews privately and protecting the participants’ identities through pseudonyms. Data management involved securing all the data in a password-protected computer which was only accessible to the first author. A professional social worker was arranged to assist with debriefing in case participants needed debriefing.

## Results

The findings of this study are presented in two forms: the socio-demographical profiles of the participants and the main research findings which are thematically presented. The researchers interviewed 12 participants of whom 6 were females and 6 were males. All participants were over the age of 18. All the participants spent a considerable length of time in the care centres with six having spent more than 10 years and the other six having spent between 2 and 8 years. After exiting care, two participants had moved to Mpumalanga and Limpopo provinces, respectively, while 10 remained in Gauteng province. One participant was in Grade 10, two were in matric and six had not been able to reach matric, although they expressed interest to do so if circumstances allowed. The remaining three participants proceeded up to tertiary institutions. All the participants were placed in care centres that are located within South Africa’s City of Ekurhuleni Metro Municipality.

### Thematic presentation of the research findings

The findings from data analysis led to five themes: the care centres as support systems; social workers as support systems; sponsors as support systems; families as support systems; and friendships as support systems.

#### Theme 1: The care centres as support systems

One of the themes that emerged from data analysis was that the CYCCs were support systems. According to the participants, they continued to receive support from CYCCs even after their departure.

Participant Zandile stated in the interview:

‘I am able to go back to the centre anytime that I have an issue or I need help. Sometimes I call them if I don’t have money to go there.’ (Participant 1, Female, 19 years old)

An interview with Sizwe also pointed to a CYCC as a source of support:

‘The centre is still supportive that’s why I’m even here in Gauteng today. There is an issue that needed to be sorted out so the people at the office sent money for transport from Mpumalanga to the centre. They also said to me if I need to talk I can let them know.’ (Participant 2, Male, 19 years old)

In another interview, Jabulani shared his experiences by saying:

‘I call my former house mother sometimes when I need encouragement. The centre is assisting me at the moment by giving me practical work experience.’ (Participant 3, Male, 20 years old)

Another participant, Lumka explained that she often visits the centre for other services:

‘I would come back to the centre to see the counsellor. I live close to the centre so I can just walk when I need help. The counsellor at the centre helped me to see how important it was for me to be there for my mother.’ (Participant 4, Female, 18 years old)

In response to the question on the available support systems, Amahle said:

‘The child and youth care workers said I could call them when I needed to talk to so that made me feel better.’ (Participant 5, Female, 23 years old)

Leeto commented that:

‘One of the child and youth care workers would say to me you will have to fend for yourself when the time comes to leave the centre.’ (Participant 6, Male, 23 years old)

What the participants reported in terms of the care centres as support systems reaffirm what previous studies found. A study of social connections and health conducted by the University of Kent (2023) has revealed that human beings are social animals who benefit from and rely on their communities in many ways. Dallas-Childs and Henderson (2020) report that the nature of the relationships for young people within and after leaving care evolve through the everyday activities. Furthermore, these relationships provide not only vital connections within the care centre but even to places and people when the time to leave care arrives (Dallas-Childs and Henderson (2020). For participants of this study, the CYCCs were the main sources of support with some specifically mentioning the crucial role played by housemothers, counsellors, child and youth care workers and other staff members.

The Systems Theory takes into account how one system can have an impact on another system, for example, leaving care centres for the youth would impact the relationships that they had with staff and affect the way they would handle issues moving forward (Schirmer & Michalakis 2019).

The Ending, which is the first stage of the Transition Model by William Bridges, involves letting go of comforts, including supportive relationships and other provisions enjoyed by the participants at the centre (Leybourne 2016). In a study conducted in the United States (US) on the challenges faced by youth who left care during COVID-19, participants expressed gratitude for the presence of staff at the care centres and even shared that they kept in touch with them (Howey, Assadollahi & Lundahl 2022). Another study focussing on the views of youth leaving care and on transition to adulthood, revealed that youth valued ongoing support from their carers (Baker 2017). This reaffirms that human beings look out to people whom they can trust and identify with when deciding on the course of action they are about to take in their lives (University of Kent 2023). The stories that were shared by our participants highlighted the appreciation that they had for the continued support from the CYCCs, with some stating that they knew that they could return to their former places of care if they had any issues or needed guidance. In the Neutral Zone stage of the Bridges’ Transition Model, individuals can experience confusion as they try to figure out how to move forward with life (Miller 2017). The participants were therefore relieved in knowing that they were welcome to go back to these centres when they needed help and guidance.

#### Theme 2: Social workers as support systems

According to the participants, social workers from some of these care centres and social workers based in communities to which they were going (their designated social workers), played an instrumental role of support.

Mark, one of the participants who was assisted by the designated social worker stated:

‘My social worker came to pick me up when I was leaving the centre. She dropped me off at the place where I was initially staying with my friends. She also said that I should contact her if I had challenges.’ (Participant 7, Male, 22 years old)

Nqcobile also explained the role of the designated social worker:

‘The social worker would do home visits just after I left the centre to see how I was doing.’ (Participant 8, Male, 21 years old)

In Natalie’s case, she was assured by a social worker that support would always be there for her whenever she needed it:

‘Before I left, the social worker said that I can get in touch with her or the counsellor if I need to.’ (Participant 9, Female, 19 years old)

Faith sharing similar sentiments to Natalie said:

‘We had help from our social workers who said that we could always come back and talk to them about stuff.’ (Participant 10, Female, 20 years old)

Amahle’s social worker helped her by sharing information on employment opportunities:

‘My social worker heard about a place that was hiring call centre agents. The company was hiring youth so that they could help them during this time of COVID-19. My social worker informed me and I went to submit my papers there. They ended up employing me.’ (Participant 5, Female, 23 years old)

As indicated from the above extracts, social workers were always available as sources of support. Participants were guaranteed social workers’ support and could go to them whenever they needed help to resolve any issue. The provision of therapy sessions by social workers was even more pivotal at the beginning of the pandemic because it was a struggle for everyone to adjust to the changes brought by COVID-19. As emerged from our findings, participants spoke about accessibility of social workers to talk about their challenges. Formal support networks involving youth’s relationships with social workers and counsellors, among others, assisted the youth to cope with the challenges that they were faced with (Pote, Swart & Carelse 2022).

Participants spoke about the therapeutic support that was provided by social workers to manage issues such as anxiety associated with COVID-19. A general sense of comfort was experienced by some participants out of knowing that social workers were accessible when needed. The findings pertaining to availability of social work support corroborated what Roberts et al. (2021) found in their study of the experiences of young people leaving care during COVID-19, that contact from social workers and some personal advisors was more important for some youth during the pandemic. The findings also concur with Cudjoe et al. (2020) who state that professionals perform a critical role of facilitating access to jobs and education for youth who have exited care. The support provided by social workers helped some participants particularly in stage three of Bridges’ Transition model (Newbery 2019). The New Beginning is when the situation starts to make sense for the individual and in the case of the participants, being linked to job opportunities by social workers helped them to start easing into their new identity of being independent adults.

#### Theme 3: Sponsors as support systems

The sponsors were among a pool of support systems available for youth who left care during the COVID-19 pandemic.

One of the participants who reflected on the supportive role of sponsors was Lumka:

‘I thought the sponsor would not cover my fees once I left the centre, but they have continued to do so.’ (Participant 4, Female, 18 years old)

Nqcobile also pointed to the supportive role of sponsors:

‘My sponsor family was there for me. When we were stuck in the house during lockdown, we would play games and cook. That’s what got me through the days, just spending time with my sponsor family.’ (Participant 8, Male, 21 years old)

Another participant, Faith said:

‘A sponsor arranged a place for me to live and helps out financially where I lack. My fees are covered by NFSAS so that helps as well.’ (Participant 10, Female, 20 years old)

Sponsors can be individuals, families, organisations or companies that commit themselves to support children and youth in care (Goebel et al. 2019). Their support is normally through contributing towards the financial and physical needs of these youth by paying for their fees, buying them clothes and toiletries, or taking them to their homes for weekend visits. As demonstrated by the participants, some sponsors offered temporary accommodation to them after exiting the care centre so that they can complete their studies in a stable environment. This supports the System Theory’s view that the environment has an influence on an individual. A stable environment provided by sponsors enables the youth to focus on their studies compared to an unstable environment, which could have the opposite effect. Davies (2022) refers to such impact of the environment on an individual as ‘positive and negative feedback loops’. A study of the experiences of youth leaving care during COVID-19 by Roberts et al. (2021) found that the pandemic brought change and disruption to young people’s education and employment. They further noticed how positive engagement in education and employment had the potential to mitigate stresses and uncertainties for these youth. Roberts et al.’s (2021) study supports the findings of this study considering the concerns raised by the participants when they were talking about their studies and how relieved they felt when their fees were paid through sponsorship. Stage one of Bridges’ Transition model describes how an individual can lose support systems, which is what some of the participants dreaded because of the sponsors’ possibility of withdrawing sponsorship (Newbery 2019). Systems Theory highlights the ripple effect from a change in one system such as leaving care, which could result in a shift in sponsorship arrangements for some of the participants (Davies 2022).

Uncertainty is an attribute experienced during stage two of the Bridges Transition Model and it was also expressed by participants such as Lumka who was not sure if her sponsor would continue to cover her fees because she left the CYCC (Leybourne 2016). Some of these concerns were justified considering that COVID-19 led to the withdrawal from commitments by most people in various sectors, including CYCCs sponsors, because it was not financially viable to honour them anymore. When youth leave the care centres, they are exposed to risks of stress associated with weakened networks of potential support (Arnau-Sabates & Gilligan 2015).

An English study conducted by Adley and Kina (2014) revealed that support networks tend to be either people whom the participants had met during their time in care or people they had met either through membership of their local churches or school. Most of the sponsors met by the participants were through their time in care and they continued to support them even after they had left care regardless of the challenges resulting from COVID-19. A South African study by Moodley, Raniga and Sewpaul (2020) focussing on the transition of youth from care, demonstrated that youth who had transitioned from care showed the importance of interdependency with host families and that sponsors played key roles in securing training, furthering education and providing employment opportunities and accommodation. Sponsors continued to reach out to the participants even after they had left the centres.

#### Theme 4: Families as support systems

Families play an instrumental role of support in both good and bad times. It was not a surprise for researchers to capture the participants’ utterances regarding the role of family as a support system.

One of the participants who pointed to the significant role of family as a support system was Zandile:

‘I had to rely on my mother from the time that I moved from the centre.’ (Participant 1, Female, 19 years old)

Zandile continued to share how her mother had been supportive:

‘My mother is always there for me when I need to talk or when I need money for something.’ (Participant 1, Female, 19 years old)

Mark described his relationship with his family:

‘I went to live in Germiston with my father and brother. In the past, we used to fight and all of that, but now it’s not like before.’ (Participant 7, Male, 22 years old)

Thabani also described his experience of family support:

‘I would call my grandmother and speak to her, I still call her. I never share with her the challenges that I face because I don’t want to worry her but just speaking to her helps.’ (Participant 11, Male, 23 years old)

Amahle explained how family support was crucial:

‘Although things were hard, we were able to go through stuff as a family.’ (Participant 5, Female, 23 years old)

As emerged from the extracts, support from families started in care for some participants who would telephonically speak to family members. What emerged from this study in relation to the family as a support system, corroborates the findings of a US study which revealed that family involvement in the life of youth in care is crucial in supporting their transition into adulthood (Cudjoe et al. 2020). Pote et al. (2022) concur with this by pointing to the importance of strong social networks for youth before they return to their families.

The support received by these youth from their families enabled them to better cope with leaving care because they had people that they could rely on for help. Some participants accepted that although the situation at home was financially difficult, they could manage because they went through it as a family. Regarding family support for youth leaving care during the COVID-19 pandemic, Kelly et al. (2021) explains how the COVID-19 crisis forced people to rely on close family support and how most of the youth leaving care needed these kinds of social networks. It was therefore not a surprise for our participants to also point to family support during the difficult times of COVID-19. Participants also highlighted the value of an opportunity afforded by COVID-19 to spend time with family members, particularly during the lockdown periods. This corroborated the findings of an Irish study by Kelly et al. (2020), which reported that the COVID-19 curfews created new opportunities for youth who had left care to spend time with their families and heal fractured relationships. Our study demonstrated that past disagreements with family members could be resolved and the interactions changed for the better. A Swedish study of youth’s ability to handle adversities after care revealed that youth who experienced problems with their families reported that contact with them provided emotional and practical support (Bengtsson, Sjoblom & Oberg 2018). In Ghana, James et al. (2017) conducted a study focussing on how youth who have been reunified with their families progressed. Their findings demonstrated high levels of hope resulting from restored relationships, guidance and support from their families. The support from families helped them to ease emotions such as fear and anxiety that were common during COVID-19 and that are popular among individuals who are in transition according to stage one of Bridges’ Transition Model (Newbery 2019).

#### Theme 5: Friendships as support systems

Another source of support for participants was friendship. The following excerpts describe the nature of support that friendships provided to participants.

Thabani explained how friends organised employment opportunities for him:

‘My friend who organised a job for me was supportive because he told me that as long as I had a job, I would have somewhere to start from.’ (Participant 11, Male, 23 years old)

In Natalie’s words, friends were helpful:

‘I can call my friends when I can, which helps.’ (Participant 9, Female, 19 years old)

Leeto had this to say:

‘I have friends who are like my brothers. One of them found me a job in Pretoria.’ (Participant 6, Male, 23 years old)

Another participant, Faith said:

‘I had friends that I could talk to as well. We were going through the same thing, so it was easy to understand each other.’ (Participant 10, Female, 20 years old)

It is not uncommon that during difficult times, families and friends become the first port of call. An English study revealed that strong connections with friends and other social groups are associated with better overall mental health and well-being (University of Kent 2023). For participants such as Faith, they could turn to friends because they were in similar circumstances and understood each other. A UK study conducted among youth in foster care by Rogers (2017) cites a participant who indicated that being friends with other youth who were in care provided a sense of belonging equated to what a family provides. Roberts et al. (2021) note that friendship can be a protective factor for youth who were in CYCCs, with the lack of friendship having the potential to isolate them as was the case during COVID-19. Participants valued friendships that they had while they were still in care, and they tried to maintain these friendships by keeping in touch. They spoke about how they keep contact with one another in order to encourage each other. Although COVID-19 curfews restricted movements, some participants were grateful for mobile phones because they could still communicate with their lived ones. This is similar to Kelly et al.’s (2020) observation regarding the ability of technology to provide ways of maintaining connections in social relations.

## Discussion

The main objective of our study was to explore availability of the support systems for youth who exited CYCCs during the COVID-19 pandemic. The findings shed light on the importance of support systems for these youth, especially at a time when they were thrust into instant adulthood while the world was in a serious pandemic (Amissah 2021). The interviews that were conducted led to the emergence of several key points.

Firstly, the relationships and connectivity are key to bettering the lives of youth who used to be in CYCCs because of the support offered (Bakketeig & Backe-Hensen, 2018; Frimpong-Monso 2020; Rogers 2018; Scottish Throughcare and Aftercare Forum 2019). Relationships that the youth established while in CYCCs were reported to be beneficial. These findings support those of Frimpong-Monso (2020), Arnau-Sabates and Arnau-Sabates and Gilligan (2015) and Sulimani-Aidan (2014) who emphasised the positive impact on youth when they have loving and caring relationships with caregivers while they are still in care and when it comes to the progress that they made after leaving care. Stage one of Bridges’ Transition Model is characterised by various emotions experienced by individuals at the thought of the possibility of losing or letting go of such relationships (Miller 2017). Although the participants experienced a sense of loss during transition, they learnt that the relationships that they built in care could be maintained by keeping in touch with caregivers and friends.

Secondly, when youth leave care centres, they need support systems to manage the transition. For the participants who left during COVID-19, their need for support was solidified because the pandemic exacerbated many of the issues already known to affect the youth (Scottish Throughcare and Aftercare Forum 2019).

Thirdly, friendships proved to be a valuable source of support for the youth. Because of the involvement of similar experiences in friendships, youth became relatable because they knew exactly how to support each other (Negard, Ulvik & Oterholm 2020). Systems Theory considers that each element of a system has an effect on the functioning of the whole which is why each support system had an effect on how the participants handled transition (Schirmer & Michalaikas 2019).

Fourthly, the recurring message from most of the participants was that of the open invitation by their former CYCCs to return for support whenever they needed. This made them to feel encouraged that they could still depend on these centres for support albeit, not to the same extent as they did when they were still residing there (Doucet 2018). The support systems that were available to the participants assisted them to figure out which steps they would take next which is what happens in stage three of Bridges’ Transition Model. Some of the participants were able to continue with their education because sponsors were willing to continue supporting them. Others were assisted by their social workers and friends to find jobs. Youth leaving care need ongoing support although of a different kind and at different times (Bakketeig & Backe-Hensen 2018). As much as the process of transition from care is a difficult one, this study showed that support systems go a long way in ensuring that the youth thrive regardless of the circumstances.

### Recommendations

Based on the research findings, the following recommendations were proposed:

- Child and youth care centres should facilitate the fostering of relationships between youth and their families when they are still in care so that they do not lose contact.
- Professionals such as social workers should ensure that youth are empowered to build healthy relationships through individual and group therapy sessions.
- Social workers should facilitate the linkage of youth to supportive resources especially when they leave these care centres.

### Study’s limitations

Interpretation and application of the findings of this study should be conducted cautiously because the study was conducted from a small sample. The findings may therefore not be applicable in other contexts.

## Conclusion

This study provided a background of the placement of youth in care centres and how relationships are built and nurtured when youth are still in care. It was revealed that these relationships are indispensable when youth exit care. The different support systems available for youth to manage transition from care centres were presented from data analysis. It was evident from the research study that youth appreciate the support that they receive after leaving care centres and that they rely on these support systems.
